# How the Venom from the Ectoparasitoid Wasp *Nasonia vitripennis* Exhibits Anti-Inflammatory Properties on Mammalian Cell Lines

**DOI:** 10.1371/journal.pone.0096825

**Published:** 2014-05-12

**Authors:** Ellen L. Danneels, Sarah Gerlo, Karen Heyninck, Kathleen Van Craenenbroeck, Karolien De Bosscher, Guy Haegeman, Dirk C. de Graaf

**Affiliations:** 1 Laboratory of Zoophysiology, Department of Physiology, Ghent University, Gent, Belgium; 2 VIB Department of Medical Protein Research, Ghent University, Gent, Belgium; 3 Laboratory for Eukaryotic Gene Expression and Signal Transduction, Department of Physiology, Ghent University, Gent, Belgium; Duke University Medical Center, United States of America

## Abstract

With more than 150,000 species, parasitoids are a large group of hymenopteran insects that inject venom into and then lay their eggs in or on other insects, eventually killing the hosts. Their venoms have evolved into different mechanisms for manipulating host immunity, physiology and behavior in such a way that enhance development of the parasitoid young. The venom from the ectoparasitoid *Nasonia vitripennis* inhibits the immune system in its host organism in order to protect their offspring from elimination. Since the major innate immune pathways in insects, the Toll and Imd pathways, are homologous to the NF-κB pathway in mammals, we were interested in whether a similar immune suppression seen in insects could be elicited in a mammalian cell system. A well characterized NF-κB reporter gene assay in fibrosarcoma cells showed a dose-dependent inhibition of NF-κB signaling caused by the venom. In line with this NF-κB inhibitory action, *N. vitripennis* venom dampened the expression of IL-6, a prototypical proinflammatory cytokine, from LPS-treated macrophages. The venom also inhibited the expression of two NF-κB target genes, IκBα and A20, that act in a negative feedback loop to prevent excessive NF-κB activity. Surprisingly, we did not detect any effect of the venom on the early events in the canonical NF-κB activation pathway, leading to NF-κB nuclear translocation, which was unaltered in venom-treated cells. The MAP kinases ERK, p38 and JNK are other crucial regulators of immune responses. We observed that venom treatment did not affect p38 and ERK activation, but induced a prolonged JNK activation. In summary, our data indicate that venom from *N. vitripennis* inhibits NF-κB signaling in mammalian cells. We identify venom-induced up regulation of the glucocorticoid receptor-regulated GILZ as a most likely molecular mediator for this inhibition.

## Introduction

Animal venoms have long been known for their inflammatory effects, for instance stings from honeybees, snakes and scorpions can induce ongoing pain and even hyperalgesia [1]–[3]. But the last few decades, there has been growing interest in the anti-inflammatory effects of these venoms. Since Billingham and colleagues have discovered the anti-inflammatory effects of honeybee venom, it has been used for the treatment of various inflammatory diseases in the oriental medicine [4]. With the aim of finding treatments for several chronic inflammatory diseases (as rheumatoid arthritis and atherosclerosis) and cancer, venom components in diverse animal groups have been screened for possible anti-inflammatory characteristics [5]–[7].

In the search for animal venom-derived immune suppressive agents, the parasitoid-host interaction is a highly intriguing system. When parasitoid wasps lay their eggs in or on a host organism (endo- or ecto-parasitoid respectively), they also inject a mixture of virulence factors that consist of ovarian and venom fluids [8]. These parasitoid fluids comprise of different active substances, exerting a large range of activities in diversified biological functions [9]. This complexity could allow for adaptation for new or widened host ranges and could increase the difficulty for a resistance to emerge considering that a multimodal tolerance might be set up by the host to insure its survival. The diverse mode of action of venom proteins from parasitoid wasps is dependent largely on their life strategy [8]. In ectoparasitoids, like for instance *N. vitripennis*, the host generally undergoes a developmental arrest and immune suppression in order to present a good environment for the development of the parasitoid offspring [10], [11]. The precise working mechanism of this immune suppression is not yet exactly characterized. But the polydnaviruses (PDVs) present in the venom of several endoparasitoid wasp species interfere with the Toll signaling pathway, which is crucial for maintaining the host immune response [12].

Toll/Imd receptors are essential for embryonic development and immunity of insects [13]. The induction of the Toll/Imd pathway by Gram-positive bacteria and fungi or Gram-negative bacteria, respectively, leads to the activation of cellular immunity as well as the systemic release of certain antimicrobial peptides. Studies in fruit flies and in mammals have revealed that the defensive strategies of invertebrates and vertebrates are highly conserved at the molecular level [14]. Infectious micro-organisms trigger activation of Toll/Imd receptors in insects or Toll-like receptors in mammals, which induces a signaling cascade culminating in the activation of NF-κB transcription factors [15]. NF-κB is the generic term for members of a family of transcription factors, that act as homo- or heterodimers and regulate transcription of multiple genes involved in immunity and inflammation. The NF-κB family in insects contains three members (Dif, Dorsal and Relish) and has expanded in mammals, which have seven NF-κB family members (p50, p105, p65/RelA, RelB and c-Rel). Different insults trigger the activation of different NF-κB dimers, which allows fine-tuning of the immune response [16]. Importantly, the NF-κB pathway is subject to tight feedback regulation, which ensures termination of NF-κB responses once the infectious danger has been eliminated [17]. NF-κB is kept inactive in resting cells by the inhibitor protein Cactus in insects and by IκB family members in mammals. When cells sense infectious danger, kinase cascades are activated that promote phosphorylation and degradation of the NF-kB inhibitor proteins, leading to NF-κB release and translocation to the nucleus, eventually activating the transcription of target genes involved in inflammation [18], [19].

Whole genome microarrays on parasitized *Drosophila melanogaster* by *Leptopilina* species revealed gene activation of Toll/NF-κB and JAK/STAT pathway components, involved in regulating immune responses toward microbes and macroparasites. The up-regulation of genes involved in these particular immune pathways suggests these hosts are better protected against microorganisms at parasitoid oviposition [20]. In endoparasitoid venoms, the PDVs encode proteins with ankyrin repeats that are also found in Cactus, the inhibitor protein of NF-κB signaling in *Drosophila*, and in mammalian IκB family members. These viral ankyrin repeats (vankyrins) however, lack the amino acid sequences necessary for their degradation/turnover. Therefore, they act as antagonists of NF-κB nuclear translocation, hence supporting parasite success. During the last decade, several PDVs in endoparasitoids have been found to express IκB-related vankyrin genes that suppress NF-κB activity during immune responses in parasitized hosts [21]–[25].

In mammalian organisms, the nuclear factor kappa B (NF-κB) transcription factors regulate important physiological processes, including inflammation and immune responses [26]. Maintenance of appropriate levels of NF-κB activity is a critical factor in immune system development and normal cell proliferation. Aberrant activation of the NF-κB pathway is involved in the pathogenesis of a number of human diseases including those related to inflammation, enhanced cellular proliferation, viral infection and genetic diseases [27], [28]. Asthma for instance is a chronic inflammation of the bronchial tubes, of which the activation of NF-κB is stimulated by agents such as allergens, ozone and viral infections [29]. NF-κB also participates in many aspects of oncogenesis, since it can suppress apoptosis and induce expression of proto-oncogenes. The constitutive NF-κB activity has been observed in a number of human cancers and the inhibition of NF-κB abrogates tumor cell proliferation [30]. Regulation and control of NF-kB activation can be a powerful therapeutic strategy for inhibiting tumor growth and viral infections and for reducing the tissue damage that follows the release of inflammatory mediators.

Various stimuli give rise to the phosphorylation and degradation of the inhibitor of NF-kB (IκB) via the IκB kinase (IKK) complex, leading to NF-κB release and translocation to the nucleus, eventually activating the transcription of target genes involved in inflammation [18]. On the other hand, activation of mitogen-activated protein kinases (MAPKs), including extracellular signal-regulated kinase 1/2 (ERK1/2), p38 and c-Jun N-terminal kinase (JNK), is another major signal transduction pathway involved in inflammation and is closely linked to the activation of the NF-κB pathway [31]. It is clear that deregulation of the transcription factor NF-κB can mediate several inflammatory diseases. Therefore several proteins tightly control its activation [32]. Among these proteins are the NF-κB inhibitory proteins, IκBα and A20, the expression of which leads to a negative-feedback response that terminates activation of NF-κB [33]. Moreover, the NF-κB inflammatory response is also modulated by endogenous glucocorticoids (GCs) that are released following a challenge with various cytokines, with the aim to restore homeostasis. These GCs are steroid hormones that can diffuse freely across the plasma membrane and bind to their intracellular receptor, the glucocorticoid receptor (GR). Because of their strong anti-inflammatory characteristics, GCs have been used since the late 1940s to treat inflammatory and autoimmune disease conditions [34].

*Nasonia vitripennis* is an ectoparasitoid wasp that prefers *Sarcophaga* flesh flies as host organism. Bioassays uncovered that the venom of this wasp causes developmental arrest, increase of lipid levels, induction of apoptosis in certain tissues and suppression of the host immune system [35]–[38]. In contrast with several endoparasitoid wasps, *N. vitripennis* only injects venom and no PDVs into the host and therefore cannot express IκB-related vankyrin genes. Interestingly, microarray analysis on parasitized *S. crassipalpis* pupae by *N. vitripennis* suggested that the venom also targets the NF-κB and MAPK pathways in the host in order to regulate the immune response [39]. Since conserved parallels have been noted between the inflammatory Toll/Imd pathways of *Drosophila,* and immune signaling pathways that activate NF-κB in mammals, we have investigated whether venom from *N. vitripennis* modulates NF-kB activation in mammalian cells.

Using a well characterized NF-κB reporter gene assay in fibrosarcoma cells [40], we found that *N. vitripennis* venom, at subcytotoxic doses, inhibits NF-κB activity. In addition, we found that *N. vitripennis* venom, in murine macrophage-like Raw264.7 cells, inhibited LPS-induced expression of the pro-inflammatory NF-κB target Interleukin-6 (IL-6). Our findings suggest that the venom-induced up-regulation of GILZ, a GR-regulated gene, is most likely the molecular mediator for this inhibition. One has to keep in mind, however, that this venom presents a highly complex mixture that, besides the 79 proteins identified [41], consists of organic molecules, amines, salts, minerals and alkaloids [42]. The unique find that the venom of an ectoparasitic wasp is able to inhibit one of the most important immune pathways in mammals, focused our research activities in unraveling the effect of the venom on this pathway in depth. This innovative study therefore, explored not only the effect of *N. vitripennis* venom on the canonical NF-κB pathway, but also the effect on the MAPK signaling pathways and negative regulatory mechanisms of NF-κB activation.

## Results

### 1. Effect of venom on NF-κB signaling

#### a. Venom inhibits NF-κB activation in TNF-stimulated L929sA fibrosarcoma cells

**Figure 1:**
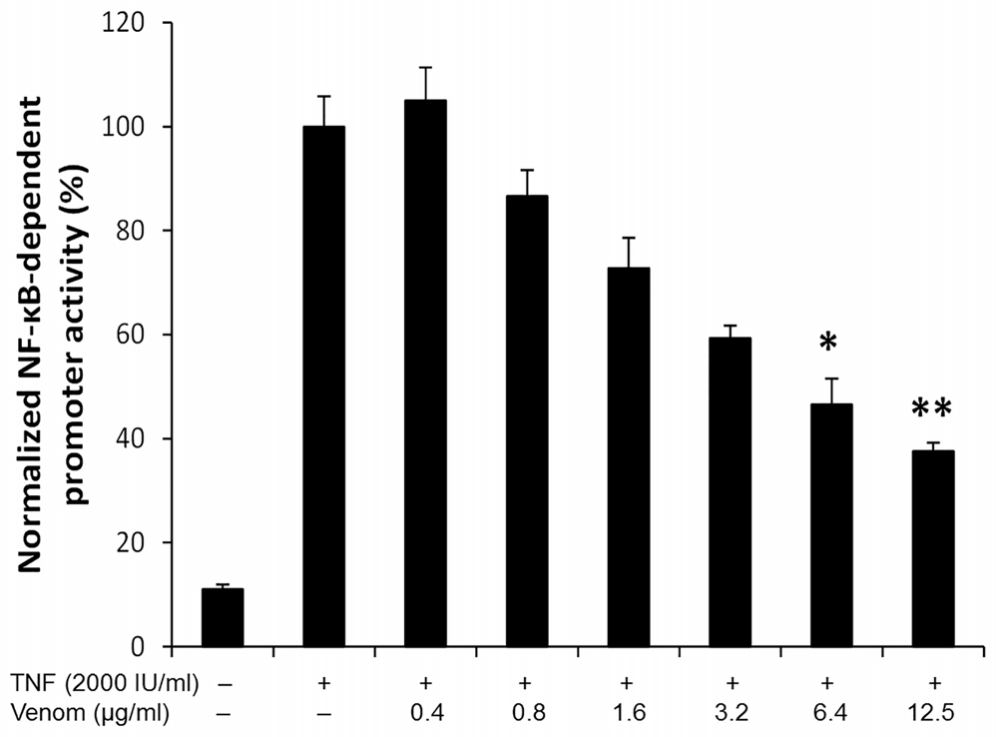
Venom from *N. vitripennis* inhibits TNF-induced expression of a NF-κB-dependent reporter gene. L929sA cells stably transfected with a NF-κB-dependent reporter gene were pretreated with indicated concentrations of venom for 15 minutes followed by stimulation for 6 hours with TNF (2000 IU/ml). Luciferase activity was normalized for β-galactosidase expression. The data are expressed as the mean ±SDs of three biological replicates. Normality was confirmed by a Shapiro-Wilk test (W = 0.9689). * p<0.05, ** p<0.01 versus TNF alone, ANOVA with Bonferroni posthoc test.

To assess the effects of *N. vitripennis* venom on NF-κB activity, we used a well characterized NF-κB reporter gene assay [40]. Briefly, the system makes use of L929sA cells stably transfected with a recombinant promoter with an NF-κB-responsive element derived from the IL-6 gene promoter (p(IL6κB)_3_50hu.IL6P-luc+), and a constitutively expressed reporter gene construct (pPGKbGeobpA), expressing β-galactosidase [43]. L929sA cells respond to TNF, followed by an induction of the NF-κB-reporter gene. Only subcytotoxic concentrations of venom were used on the cells (see figure S1 for MTT cell viability assay). As shown in figure 1, enhanced luciferase expression levels were measured in response to TNF, whereas pretreatment with venom was found to potently inhibit reporter gene expression in a dose-dependent manner (see figure S2 for the separate luciferase and β–galactosidase read-outs of the NF-kB-reporter assay). Similar assays were performed on L929sA cells stably transfected with a recombinant promoter with either the CRE (cyclic AMP response element) responsive element or the AP-1 responsive element, which confirmed the specificity of the venom towards NF-kB (see figure S3).

#### b. Venom inhibits IL-6 mRNA and protein expression in LPS-stimulated Raw264.7 macrophages

**Figure 2:**
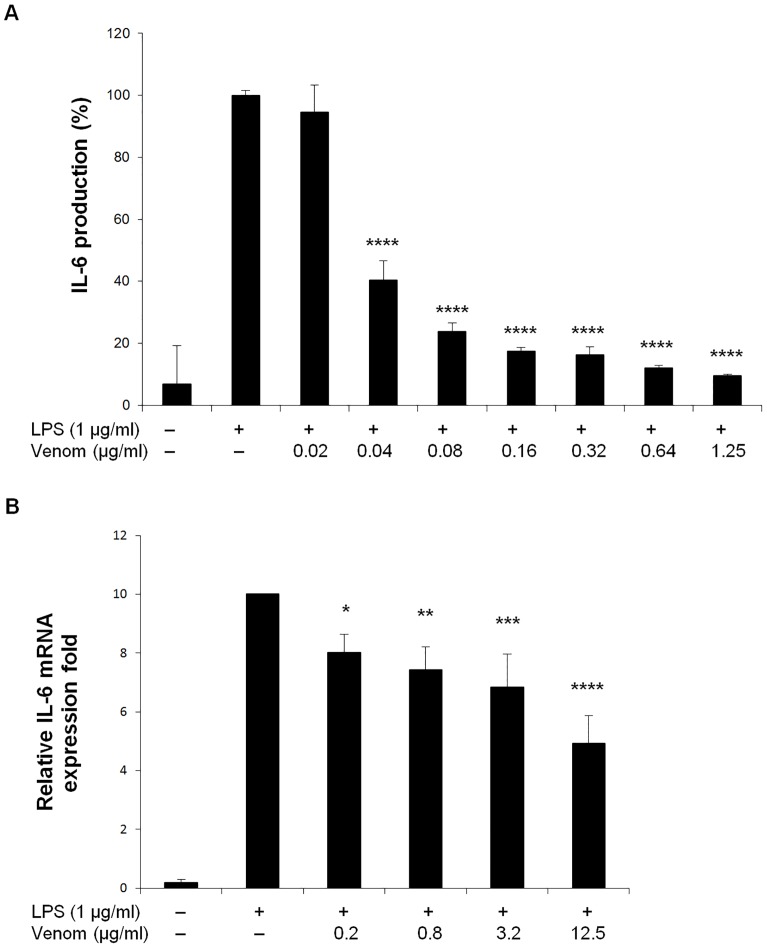
Effect of *N. vitripennis* venom on IL-6 production in LPS-stimulated Raw264.7 cells. (A) Cells were stimulated with LPS (1 µg/ml) for 6 hours, alone or after 15 minutes pre-incubation with various concentrations of venom. IL-6 produced and released into the culture medium was assayed by ELISA. (B) Cells were stimulated with LPS (1 µg/ml) alone or with indicated amount of venom for 6 hours. Total RNA was isolated and mRNA expression level of IL-6 was determined by qRT-PCR. hPRT1 and cyclophilin were used to normalize the data. The data are expressed as the mean ±S.D. of three biological replicates. Normality was confirmed by a Shapiro-Wilk test (W = 0.7617 for A; W = 0.8673 for B). * p<0.05, ** p<0.01, *** p<0.001, **** p<0.0001 versus LPS alone, ANOVA with Bonferroni posthoc test.

Interleukin (IL)-6 is a pleiotropic cytokine involved in acute phase and immune reactions and inflammatory responses, the expression of which in response to inflammatory stimuli, strongly depends on NF-κB [44]. Macrophages are key players in the inflammatory response, and they secrete high levels of IL-6 upon ligation of Toll-like receptors (TLRs) by pathogen-associated molecular patterns (PAMPs). We therefore explored the effect of *N. vitripennis* venom on TLR4-dependent, LPS-induced IL-6 production in Raw264.7 mouse macrophages. As shown in figure 2A, the elevated levels of IL-6 protein detected after 6 hours LPS treatment were strongly repressed in the presence of the venom in a concentration-dependent manner. *Nasonia vitripennis* venom pretreatment also inhibited LPS-induced IL-6 mRNA levels, yet this effect was less prominent and required ten-fold higher doses of venom (figure 2B).

#### c. Venom does not interfere with canonical NF-κB signaling in the cytoplasm

**Figure 3:**
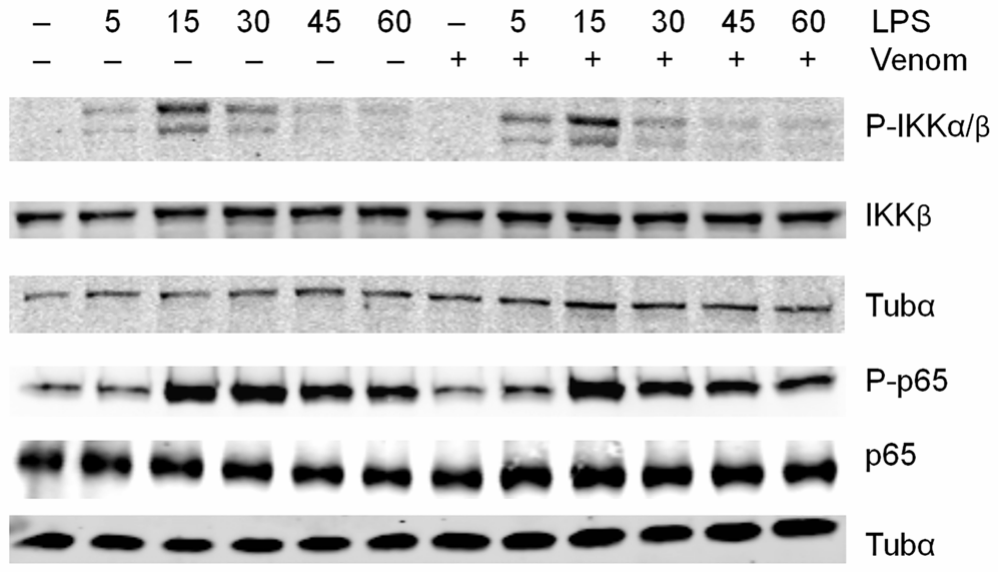
Effect of venom on cytosolic protein activity. Raw264.7 cells were left untreated or were pretreated with 5 µg/ml venom for 15 minutes and then stimulated with 1 µg/ml LPS for the indicated times. Total cell extracts were assayed by Western blot analysis using antibodies against indicated proteins. The unphosphorylated protein and Tubulin-α were used as loading controls. A representative result from three separate experiments is shown.

One of the major mechanisms involved in the transcriptional activation of NF-κB is the activation of the IKK kinase complex and the concomitant phosphorylation and degradation of the IκBα protein, which allows the release of free NF-κB and its subsequent translocation to the nucleus. Furthermore, post-translational modifications of p65 have been demonstrated to be critical for p65-driven gene expression of many target genes [45]. The IKK complex is known to phosphorylate p65 at serine 536 [46], in this way regulating p65 nuclear localization. We assessed whether *N. vitripennis* venom affects these early LPS-induced events in Raw264.7 cells (figure 3). We found that LPS promoted phosphorylation of IKK in its activation loop that was apparent after 5 minutes and lasted up to 15 minutes of LPS treatment. Venom pretreatment did not affect IKK phosphorylation. Next, it was investigated whether the venom alters LPS-induced phosphorylation of p65. As the Western blot analysis in figure 3 shows, p65 phosphorylation in Raw264.7 cells is highly increased after 15 minutes LPS induction. However, no difference in LPS-induced p65 phosphorylation was apparent after venom co-treatment (figure S4).

#### d. Venom does not interfere with nuclear translocation of the p65 subunit

**Figure 4:**
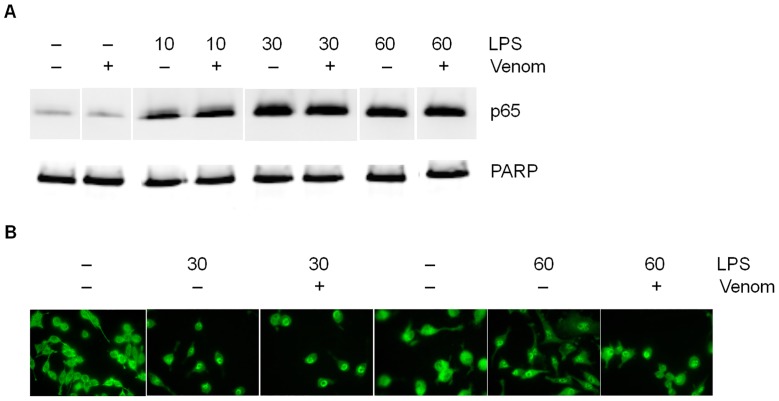
Effect of venom on nuclear translocation of p65. A. Western blot analysis of nuclear extracts from Raw264.7. After pre-incubation with *N. vitripennis* venom (5 µg/ml) for 15 minutes, cells were induced for the indicated times with LPS (1 µg/ml). Subsequently, nuclear extracts were subjected to Western blot analysis to determine p65 levels. Separation of nuclear and cytoplasmic fractions was verified using PARP as control for the nuclear fractions. B. After 15 minutes pre-incubation with *N. vitripennis* venom, cells were induced for the indicated times with LPS (1 µg/ml). Immunofluorescence staining was performed to visualize the trafficking of the p65 subunit. Representative results from three separate experiments are shown.

LPS induction in Raw264.7 macrophage cells leads to canonical NF-κB activation, that is followed by nuclear translocation of the NF-κB p65 subunit prior to NF-κB/DNA binding. Therefore, the level of p65 was measured in nuclear extracts of Raw264.7 cells either untreated or venom-treated for 10, 30 or 60 minutes LPS induction. Antibody against Poly ADP-Ribose polymerase (PARP) was used as a loading control for the nuclear fractions. When analyzed by Western blotting, LPS-induced p65 nuclear translocation was not affected by *N. vitripennis* venom (figure 4A). These results were confirmed in figure 4B by immunofluorescence staining to visualize the trafficking of the p65 subunit. LPS induction after 30 and 60 minutes caused an accumulation of green fluorescent signal in the nucleus, conform the translocation of p65 from the cytoplasm to the nucleus. Co-incubation of these cells with venom, however, did not alter this translocation.

#### e. Venom does not interfere with NF-κB transactivation independent from the promoter context

Since no influence of *N. vitripennis* venom was apparent on the subcellular localization of p65 NF-κB following inflammatory stimuli, the next step was to investigate whether the observed inhibition of NF-κB activity (figure 1) could be explained by a nuclear phenomenon. To approach this question, it was investigated whether *N. vitripennis* venom could interfere with NF-κB transactivation, independent from the promoter context. Therefore, its effect on the transcriptional activation of a Gal4-p65-dependent reporter gene, transactivated by the p65 chimera, was analyzed. This Gal4-p65 fusion protein, composed of the DNA-binding domain of the yeast nuclear protein Gal4 and the transactivation domain of the NF-κB subunit p65, stimulates p(Gal)_2_-50hu.IL6P-luc+, a luciferase reporter gene, preceded by two Gal4-binding DNA sequence elements and the minimal IL-6 promoter. In this particular nuclear setup, no direct influence of other responsive element-bound transcription factors, which are normally present in the IL-6 promoter context, or interference of cytoplasmic events need to be taken into account. When the glucocorticoid receptor (GR), a ligand-activated transcription factor present in the cytoplasm, binds to its agonist, e.g. the synthetic glucocorticoid dexamethasone (DEX), the receptor translocates to the nucleus and interferes with the transactivating function of NF-κB, in a process called transrepression. Cells were incubated with DEX as a positive control for transrepression of the NF-κB pathway.

**Figure 5:**
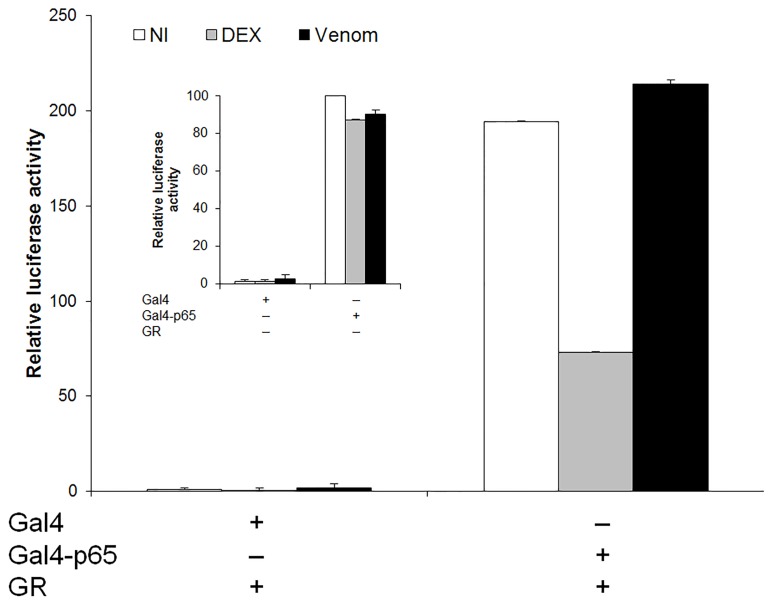
Effect of *N. vitripennis* venom on the Gal4-p65-mediated expression of a reporter gene in a Gal4 one-hybrid setup. HEK293T cells were transiently transfected by the PEI method with 100(Gal)2-50.huIL6P-luc+ and with various expression plasmids, the total amount of DNA being fixed at 250 ng, i.e., pGal4 (10 ng) or pGal4-p65 (10 ng) whether or not with pGR (25 ng). 10^−6^ M DEX or 5 µg/ml of venom was added 24 hours before analysis. Lysates were made and the relative luciferase activity was determined by using β-gal values as a basis for normalization. The data are expressed as the mean ±S.D. of three biological replicates.

In these experiments, HEK293T cells that are devoid of GR, were transiently transfected with the expression constructs, and treated with venom, DEX or were left untreated. Finally, GAL4-p65 driven expression of luciferase was quantified based on the measurement of its activity. Whereas Gal4 protein on its own cannot activate the reporter, overexpression of the nuclear fusion protein Gal4-p65 clearly activated reporter gene expression, as expected (figure 5). After adding DEX to the cells, the level of transactivation by the nuclear fusion protein Gal4-p65 could be specifically repressed, when sufficient exogenous GR was present. However, repression of Gal4-p65-induced transcriptional activation could not be achieved by the *N. vitripennis* venom, indicating that compounds in the venom do not interfere with the transactivation potential of the p65 subunit in the nucleus or that they act on specific components of the transcriptional machinery that is not involved in this Gal4 assay.

### 2. Effect of venom on MAPK activation pathways: venom causes prolonged JNK-activation

**Figure 6:**
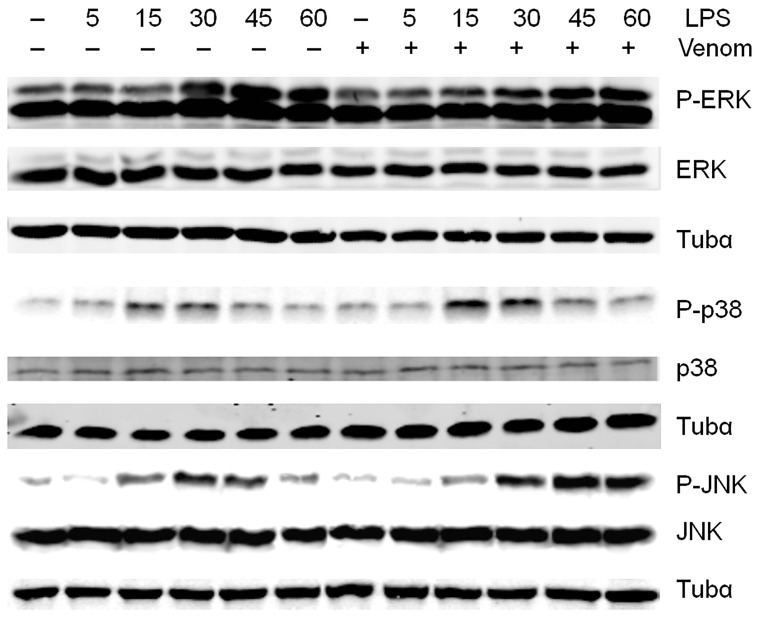
Effect of *N. vitripennis* venom on LPS-induced phosphorylation of MAPKs. Raw264.7 cells were either left untreated or treated with venom (5 µg/ml) for 15 minutes followed by stimulation with 1 µg/ml LPS for the indicated times. Total cell extracts were assayed by Western blot analysis using the indicated antibodies. Tubulin-α and the respective unphosphorylated proteins were used as loading controls. A representative result from three separate experiments is shown.

It is well established that the phosphorylation and, in turn, activation of MAPKs, i.e. p38, ERK1/2 and JNK, are crucial for LPS-induced AP-1 and NF-κB activation and subsequent up-regulation of pro-inflammatory mediators in macrophages [47]. p38 MAP kinase and/or ERK are required for NF-κB activation in response to LPS and JNK is essential for activation of transcription factor AP-1 [48]–[50]. To investigate whether regulation of the activation of these MAPKs is involved in the molecular mechanism of action by *N. vitripennis* venom, phosphorylation of these MAPKs was further examined. Hereto, Raw264.7 cells were pretreated with venom followed by addition of LPS for different time points. Whole-cell lysates were prepared and the total expression of these different MAP kinases as well as levels of phosphorylated MAPKs were determined by Western blot analysis. The results showed LPS stimulation activates MAPKs in a time-dependent manner. Pretreatment with venom had no effect on the LPS-induced phosphorylation of ERK1/2 and p38 (figure 6 and figure S5). However, after 60 minutes LPS induction, prolonged JNK activation could be observed when *N. vitripennis* venom was added to the cells.

### 3. Effect of venom on NF-κB regulated secondary anti-inflammatory processes

**Figure 7:**
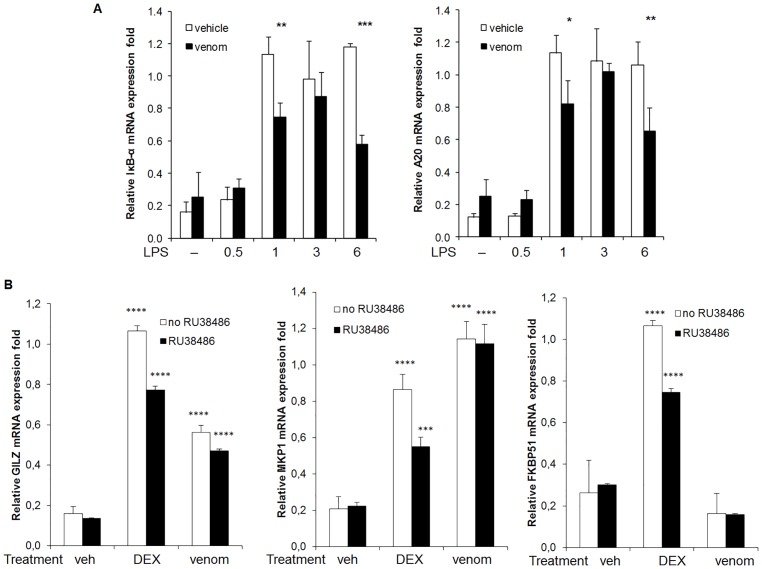
Effect of *N. vitripennis* venom on the mRNA expression of secondary anti-inflammatory processes. A. Raw264.7 cells were stimulated with LPS (1 µ/ml) alone or with 5 µg/ml venom for 30 minutes, 1 hour, 3 or 6 hours. Total RNA was isolated and the mRNA expression of IκBα and A20 was determined by qRT-PCR. hPRT1 and cyclophilin were used to normalize the data. The data are expressed as the mean ±S.D. of three biological replicates. Normality was confirmed by a Shapiro-Wilk test (W = 0.9131 for IκBα; W = 0.8630 for A20). * p<0.05, ** p<0.01, *** p<0.001 for the LPS group compared with the respective LPS and venom group, ANOVA with Bonferroni posthoc test. B. Raw264.7 cells were pre-incubated with RU38486 for 1 hour when indicated and then stimulated with 10-6 M DEX or with 5 µg/ml venom for 6 hours. Total RNA was isolated and the mRNA expression of GILZ, MKP1 and FKBP5 was determined by qRT-PCR. hPRT1 and cyclophilin were used to normalize the data. The data are expressed as the mean ±S.D. of three biological replicates. Normality was confirmed by a Shapiro-Wilk test (W = 0.8952 for GILZ; W = 0.8834 for MKP1; W = 0.8424 for FKBP5). *** p<0.001, **** p<0.0001 for the DEX or venom group compared with the respective vehicle group and for the DEX + RU38486 or venom + RU38486 group compared with the respective RU38486 group, ANOVA with Bonferroni posthoc test.

Next to the well-studied direct regulation of NF-κB signaling, the regulatory networks that control the NF-κB response are extensive. The best studied and well accepted mechanism for termination of the NF-κB response involves the resynthesis of IκBα proteins induced by activated NF-κB [51]. Newly synthesized IκBα can enter the nucleus, remove NF-κB from the DNA, and relocalize it to the cytosol [52]. Another important negative regulator of the NF-κB response is A20, which is up-regulated following NF-κB activation. Subsequently, A20 down regulates NF-κB through its dual function as a deubiquitinase and ubiquitin ligase [53]. Furthermore, glucocorticoids (GCs) are also negative regulators of NF-κB activation and are widely used as anti-inflammatory agents [54]. GCs diffuse through the cell membrane and bind to their inactive cytosolic receptors (GRs), which then undergo conformational modifications that allow for their nuclear translocation. In the nucleus, activated GRs modulate transcriptional events by directly associating with DNA elements. In addition, activated GRs also act by antagonizing the activity of transcription factors, in particular NF-κB, by direct and indirect mechanisms [55]. For these reasons, we were interested whether the venom from *N. vitripennis* causes any differences in these secondary regulatory mechanisms of the NF-κB response. We first tackled this question by looking at the mRNA expression of two immediate-early genes, IκBα and A20 (figure 7A), and of three GR-regulated genes, glucocorticoid inducible leucine zipper (GILZ), mitogen-activated protein kinase (MAPK) phosphatase-1 (MKP1), and FK506 binding protein 51 (FKBP5) (figure 7B). To study the IκBα and A20 mRNA expression, Raw264.7 cells were either pretreated with venom or with insect saline buffer (ISB) as a control and subsequently stimulated with LPS for 30 minutes, 1 hour, 3 or 6 hours. Then, RNA was isolated from the cells, reverse-transcribed and analyzed by real-time PCR. As shown in figure 7A, mRNA expression of IκBα and A20 is up-regulated after 1 hour of LPS induction and remains elevated for 6 hours after LPS induction. When venom is applied to the cells, both IκBα and A20 expression are significantly suppressed after 1 hour and after 6 hours LPS induction. Next, the transcriptional response on several GR-regulated genes was investigated. The synthetic ligand of GR, dexamethasone (DEX) was used as a positive control and the GR antagonist RU38486 was used to monitor whether the ligand-binding domain of GR was affected by the venom. Raw264.7 cells were pre-incubated with RU38486 for 1 hour when indicated and then stimulated with DEX or with venom for 6 hours. As the results in figure 7B show, the transactivation by DEX causes an increase in all three tested genes, while the prior addition of the antagonist resulted in a decrease of their expression, as expected. Interestingly, when *N. vitripennis* venom was added to the cells, an increase in GILZ expression could also be seen, halfmaximal to the increase caused by DEX. Notably, venom-induced MKP1 expression was even higher than the response to DEX. However, no effect of venom was apparent on FKBP5 expression. RU38486 addition prior to venom did not affect gene expression of these three GC indicator genes.

**Figure 8:**
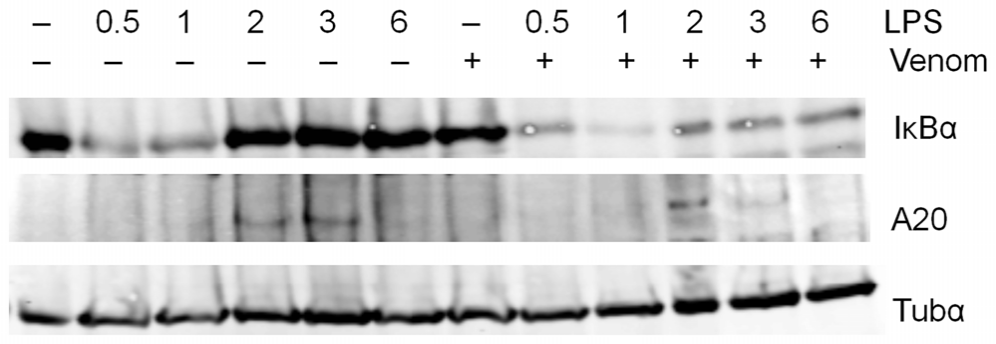
Effect of venom on IκBα and A20 protein levels. Raw264.7 cells were left untreated or were pretreated with 5 µg/ml venom for 15 minutes and then stimulated with 1 µg/ml LPS for the indicated times. Total cell extracts were assayed by Western blot analysis using antibodies against indicated proteins. Tubulin-α was used as a loading control. A representative result from three separate experiments is shown.

Since venom suppressed IκBα and A20 mRNA expression, we were interested whether this was concomitant with a suppression at the protein level. Therefore, Raw264.7 cells either or not pretreated with *N. vitripennis* venom, were treated with LPS for 30 minutes, 1 hour, 2, 3 or 6 hours (figure 8 and figure S6). Western blot analysis showed that IκBα is almost completely degraded after 30 minutes and its resynthesis was detectable after 2 hours of LPS induction. When venom was applied on the cells, the resynthesis of IκBα remains suppressed even after 6 hours of LPS induction. This probably means that the lowered amount of IκBα mRNA caused by the venom (figure 7A) resulted in less translated IκBα protein. When looking at the presence of A20, an elevation at the protein level was noticed after 2 and 3 hours LPS induction. Venom caused a slight elevation of A20 after 2 hours of LPS induction, but a significant suppression could be noted when LPS was applied to the cells for 3 hours. This is probably the result of the suppressed A20 mRNA expression (figure 7A).

## Discussion

Studies on the immune suppressive effect of *N. vitripennis* venom have always been directed on hemocytes of their hosts [56], [57] or cultured insect cells [37] [56] [58]–[60]. While investigating the possible function of parasitoid venom on mammalian cells may seem unlogic at first, the plethora of available research tools in these cell lines, that highly exceeds what is available in insect cell lines, convinced us to start working on mammalian cell lines. Secondly, NF-κB transcription factors, the MAPK signal transduction pathways and the glucocorticoid receptor signaling pathway that were studied, are evolutionary conserved in metazoan animals [61]–[65]. Finally, there is an enormous request for anti-inflammatory compounds in medicine that represents the high amount of future applications available [66]. Since so many diseases are driven by chronic inflammatory responses, finding good drugs to control this excessive immune response has been the focus of much research. The last few decades, venoms have also been added to the growing list of anti-inflammatory drugs [67]. The extraordinary finding that the venom of a parasitoid wasp is able to suppress the major mammalian immune pathway, intrigued us to fully disentangle this interaction. Respectively, we are the first to investigate the inhibitory effect of *N. vitripennis* venom on the NF-κB pathway in mammalian cells and to suggest its mode of action.

**Figure 9:**
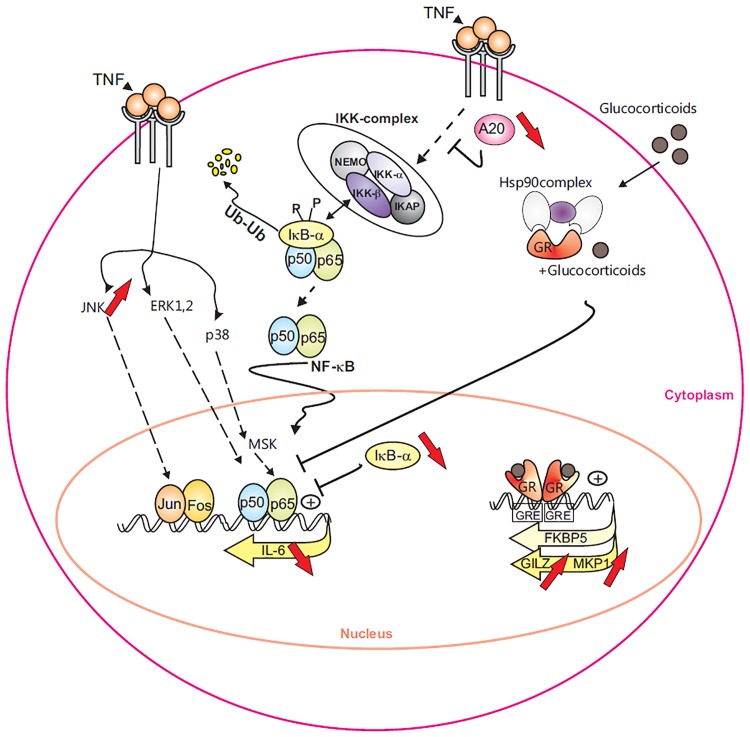
Proposed model of venom interference with the NF-κB and glucocorticoid signaling pathways. TNF stimulation leads to activation of the canonical NF-κB signal transduction pathway marked by IκBα degradation and translocation of the p65-p50 dimer to the nucleus. Subsequent NF-κB DNA binding enables transcription of different genes, among which the cytokine IL-6. Venom treatment leads to the inhibition of TNF induced IL-6 gene expression. In addition, TNF activates the MAPKs,JNK, ERK1/2 and p38, and pretreatment with venom results in prolonged JNK activation. The negative NF-κB regulators A20 and IκBα are indicated and both were suppressed by the venom. Glucocorticoid binding to the cytosolic Glucocorticoid Receptor (GR) results in the dissociation of chaperoning proteins, followed by GR translocation to the nucleus, where it can interfere with the activity of NF-κB. Activated GR can also directly bind to the DNA, stimulating transcription of FKBP5, MKP1 and GILZ target genes. Only the two latter genes are transcriptionally induced by the venom. The red arrows mark the various levels at which venom of *N. vitripennis* interferes with the represented cellular signaling pathways.

Previous experiments on natural host organisms have shown that maternally derived venom from *N. vitripennis* disrupts host immune responses almost immediately following oviposition [38]. Microarray analysis on parasitized *S. crassipalpis* pupae by *N. vitripennis* already suggested NF-κB and MAPK pathways in the host as potential candidates for the regulation of the immune response to envenomation [39]. The current study was conducted to investigate the potential anti-inflammatory and anti-NF-κB responses of *N. vitripennis* venom on mammalian cells. A proposed model of the interactions between the venom and the NF-κB pathway is illustrated in figure 9. Since this venom has a high cytotoxicity to certain insect cell types [68], cell viability was tested to establish the appropriate concentration ranges of venom for the analysis of ongoing experiments. When incubation periods of 6 hours were applied, 5 or 10 µg/ml venom appeared to be non-toxic concentrations and were used in further experiments. Our initial focus was to see whether *N. vitripennis* venom could inhibit the NF-κB immune signaling pathway. This was first studied in murine fibrosarcoma cells stably transfected with a recombinant promoter that has an NF-κB-responsive element. Interestingly, when these cells were incubated with *N. vitripennis* venom, a clear dose-related inhibition of relative luciferase activity could be noted. Venom-mediated suppression of the NF-κB pathway could be confirmed in Raw264.7 macrophages, since the venom dose-dependently suppressed IL-6 mRNA and protein expression. This is a remarkable result, since this is the first report that venom from an ectoparasitic wasp is able to suppress NF-κB signaling in mammalian cell lines.

The following study focused on localizing the intrusion step by venom compounds. In 2006, Gilmore and Herscovitch reported on the identification of more than 785 inhibitors of NF-κB signaling [69]. They noted that in most cases, inhibition is based on a given agent's ability to block one or more steps of NF-κB signaling in a tissue culture-based system after stimulation of resting cells with an NF-κB inducer, most commonly either TNF of LPS. More than half of those described NF-κB inhibitors block a signal before the NF-κB dimer is able to bind the DNA in the nucleus. *Nasonia vitripennis* venom however, did not cause an effect on the cytosolic events, nor on the translocation of the NF-κB dimer to the nucleus for binding to the DNA, nor did it disturb the transactivation function of p65. These intriguing results forced our research activities into the direction of other pathways or regulatory mechanisms that could result into this inhibition of NF-κB activity.

The phosphorylation and activation of three major MAPKs – p38, ERK1/2 and JNK – have been shown to initiate inflammatory gene expression in LPS-induced macrophages. Therefore, the effect of venom on these pathways was also investigated. Interestingly, we found that venom prolonged LPS-induced JNK activation in Raw264.7 macrophages. Genotoxic stresses, e.g. UV or γ-irradiation, and TNF-induced reactive oxygen species (ROS) accumulation induce long-lasting or prolonged MAPK activation, which in turn contributes to cell death [70]. NF-κB on the other hand, can protect cells against TNF cytotoxicity by inhibiting the sustained phase of TNF-induced JNK activation [71]. Prolonged JNK activation as a result of venom induction could be the result of NF-κB inhibition and eventually even lead to cell death. Indeed, *N. vitripennis* venom induces apoptosis of insect cells [72], while in murine macrophages, only high doses and long induction times were cytotoxic to the cells.

Since aberrant NF-κB activity can directly lead to uncontrolled tissue damage and deleterious disease [73], [74], the NF-κB driven inflammatory responses need to be down-regulated and properly terminated. Therefore, many distinct negative regulatory feedback mechanisms have evolved that operate at different molecular levels in the NF-κB signaling pathway [17]. For instance, several proteins among which IκBα and A20, are NF-κB target genes, constituting a negative-feedback response. *Nasonia vitripennis* venom was, by causing suppression of inhibitors of NF-κB signaling, indirectly found to give rise to an elimination of this negative-feedback response. The abrogation of this negative regulation can therefore be seen as the normal consequence of the venom-induced suppression of the NF-κB activity. Another regulation of the NF-κB activation is realized by glucocorticoids [75]. Today, nothing is known about possible steroids or corticoid-like substances in *N. vitripennis* venom. However, besides peptides and proteins, venoms are known to contain organic molecules, amines, alkaloids, salts and minerals [76] and recently, the venom from the toad *Bufo bufo gargarizans* was found to contain steroids that displayed potent inhibitory activities against cancer cells [77]. When we looked at genes that are regulated by GR, *N. vitripennis* venom induction caused an up-regulation of GILZ and MKP1. Intriguingly, GILZ and MKP1 are known to inhibit NF-κB signaling; GILZ through the interaction with p65 [78], and MKP1 via dephosphorylation and inactivation of members of MAPK family, preferably JNK and p38 MAPK [79]. Studies in T lymphocytes demonstrated that GILZ reduced NF-κB transcriptional activity [80]. Subsequent in vivo studies showed that administration of a GILZ peptide inhibits disease development in the experimental autoimmune encephalomyelitis model of multiple sclerosis, in part via effects on T cell activation [81]. Together, these findings demonstrate a suppressive role of GILZ in the regulation of immune cell activation. We speculate GILZ up-regulation as the most likely candidate for the venom-induced effect, but the precise mode of action still needs to be elucidated.

We can conclude that in analogy with inhibition of immune responses in the natural insect host, also in mammalian cells anti-inflammatory responses of the venom can be observed. However, an interesting difference concerning MAPK pathways was observed. While the microarray study on the insect host showed significant down-regulation of a MAPK gene and up-regulation of a MAPK-phosphatase, an increase in activation of the JNK pathway was seen in the mammalian system. Apparently, the venom has a different mode of action in insects than in mammalian cells, which seems interesting to investigate more into detail.

In view of the complexity of the NF-κB pathway, the identification of specific inhibitors with acceptable side-effects has been challenging, and new molecules are still actively sought after. Perhaps, venom from the ectoparasitoid wasp *N. vitripennis* can meet with these conditions by looking at the results presented here, but for sure, future experiments need to explore this further.

## Materials and Methods

### 1. Isolation of crude wasp venom

*Nasonia vitripennis* wasps were reared on pupae of the flesh fly, *Sarcophaga crassipalpis*, and maintained at 25°C with a daily 15∶9 light: dark cycle. Female wasps were allowed to host feed on flesh fly pupae for 24 hours. Venom gland reservoirs were dissected into insect saline buffer (ISB) [150 mM NaCl, 10 mM KCl, 4 mM CaCl_2_, 2 mM MgCl_2_, 10 mM Hepes] [82] and centrifuged at 12,000×g for 10 minutes at 4°C. The supernatant containing the venom was transferred to a clean microcentrifuge tube and stored frozen at −70°C. Total protein in crude venom was determined colorimetrically at 595 nm using a Coomassie Protein Assay Reagent (no 23200, Thermo Scientific).

### 2. Chemicals

The origin and activity of TNF, as well as the preparation of luciferase (luc) reagent, were described previously [43]. Dexamethasone (DEX), LPS from *Escherichia coli*, mouse anti-tubulin-α and the specific GR antagonist RU38486 were purchased from Sigma Chemical Co. (St. Louis, MO). Antibodies to rabbit IκBα (C-21), mouse A20 (A-12), rabbit PARP-1/2 (H-250 and rabbit NF-κB p65 (C-20) were obtained from Santa Cruz Biotechnology (Santa Cruz, CA, USA). Antibodies to rabbit phospho-IKKα (ser180)/(ser181)β, rabbit phospho-NF-κB p65(ser536), rabbit phospho-ERK MAPK and rabbit phospho-p38 MAPK were purchased from Cell Signaling Technology (Danvers, MA, USA). Corresponding fluorescent-coupled secondary antibodies were purchased from Rockland, Gilbertsville, PA.

### 3. Cell culture

Raw264.7 cells, a mouse macrophage-like cell line, were maintained in RPMI 1640 medium, murine fibrosarcoma L929sA cells and human embryonic kidney (HEK) 293T cells were maintained in DMEM at 37°C in 5% CO_2_ humidified air. Both media were supplemented with 10% fetal bovine serum, 100 units/ml penicillin and 0.1 mg/ml streptomycin. Twenty-four hours before induction, cells were seeded in multiwell dishes such that they were confluent at the time of the experiment.

### 4. Plasmids and transfection procedure

pGal4, pGal4-p65 and p(Gal)_2_-50hu.IL6P-luc+ were previously described [83]. pPGKβgeobpA was a gift from P. Soriano (Fred Hutchinson Cancer Research Center, Seattle). p(IL6κB)_3_50hu.IL6P-luc+ [40] and p(GRE)_2_-50-luc [84] were described previously. Transient transfections were performed using polyethylenimine (PEI) [85]. Stable transfection was performed by the calcium phosphate precipitation procedure according to standard protocols [43].

### 5. Reporter gene analysis

Luciferase (luc) and galactosidase reporter assays were carried out according to the manufacturer's instructions (Promega) and have been described previously [43]. Luc activity, expressed in arbitrary light units, was corrected for differences in protein concentration and transfection efficiency in the sample by normalization to constitutive β-gal levels. Β-gal levels were quantified with a chemiluminescent reporter assay Galacto-Light kit (TROPIX).

### 6. Enzyme-linked immunosorbent assay of IL-6

Murine IL-6 ELISA was performed using the Mouse IL-6 CytoSet from Invitrogen.

### 7. Western blot analysis

After inductions (indicated in the figure's legends) or solvent controls where appropriate, cells were washed with ice-cold phosphate-buffered saline (PBS), harvested with a rubber policeman and collected in 1 ml of PBS by centrifugation for 10 minutes at 500 g (4°C). Preparation of cytoplasmic or nuclear cell extracts have been described previously [40]. If total cell lysates were used, cells were lysed in SDS sample buffer [62.5 mM Tris-HCl, pH 7.5, 2% (w/v) SDS, 10% glycerol, 50 mM dithiothreitol (DTT), and 0.01% (w/v) bromophenol blue]. To shear DNA and reduce sample viscosity, lysates were sonicated for 1 minute in a water bath sonicator and then heated to 95°C for 5 minutes after which they were immediately cooled in ice and microcentrifuged for 5 minutes. The lysates were separated by 10% SDS-PAGE and electrotransferred onto a nitrocellulose membrane. Blots were probed using the appropriate antibodies (1∶1,000), and their corresponding immunoreactive protein (1∶10,000) was detected on an Odyssey imaging system (LI-COR Biosciences, Lincoln, USA).

### 8. RNA analysis

RNA was isolated by using TRIzol Reagent (Invitrogen) as described previously [84]. Total RNA was reverse-transcribed into cDNA using the Revert Aid H Minus First Strand cDNA Synthesis Kit (Fermentas) following the manufacturer's instructions. The expression of the genes was determined by qRT-PCR in a CFX96 Real-Time PCR Detection System (BioRad, Hercules, CA, USA) using the SYBR Green mastermix (Invitrogen). The following mouse-specific primers were used:

IL-6 forward 5′-GAGGATACCACTCCCAACAGACC-3′;

IL-6 reverse 5′-AAGTGCATCATCGTTGTTCATACA-3′;

IκBα forward 5′-TGAAGGACGAGGAGTACGAGC-3′;

IκBα reverse 5′-TTCGTGGATGATTGCCAAGTG-3′;

A20 forward 5′-CCTTGCTTTGAGTCAGGCTGT-3′;

A20 reverse 5′-TAAGGAGAAGCACGAAACATCGA-3′;

FKBP5 forward 5′-TGAGGGCACCAGTAACAATGG-3′;

FKBP5 reverse 5′-CAACATCCCTTTGTAGTGGACAT-3′;

MKP1 forward 5′-GAGCTGTGCAGCAAACAGTC-3′;

MKP1 reverse 5′-CTTCCGAGAAGCGTGATAGG-3′;

GILZ forward 5′-CCAGTGTGCTCCAGAAAGTGTAAG-3′;

GILZ reverse 5′-AGAAGGCTCATTTGGCTCAATCTC-3′.

An average of the following household genes was used for normalization:

cyclophilin forward 5′-GCATACGGGTCCTGGCATCTTGTCC-3′;

cyclophilin reverse 5′-ATGGTGATCTTCTTGCTGGTCCTTGC-3′;

hPRT forward 5′-CCTAAGATGAGCGCAAGTTGAA-3′;

hPRT reverse 5′-CCACAGGACTAGAACACCTGCTAA-3′.

The thermal cycling conditions were as follows: 5 minutes at 50°C, 2 minutes at 95°C and then 40 cycles of 20 seconds at 95°C and 40 seconds at 60°C. Melting curve analysis was used to determine primer specificity.

### 9. Immunofluorescence staining

Cells were seeded on coverslips and grown for 24 hours. Fixation, permeabilization, and staining were performed as described previously [84]. Presence of p65 was visualized with a 1∶200 dilution of anti-p65 antibody, followed by probing with a 1∶800 dilution of Alexa Fluor 488–conjugated goat anti-rabbit IgG. Staining with 4,6-diamidino-2-phenylindole (DAPI) was used for visualization of the cell nuclei. A Zeiss Axiovert 200M microscope was used for visualization of the images, and data were analyzed using Carl Zeiss Axiovision software (Carl Zeiss Instruments, Jena, Germany).

### 10. Statistical analysis

All assays were carried out in triplicate from three biological repeats and results were expressed as the mean ±SDs. Prior to statistical tests, normality of the data was confirmed by Shapiro-Wilk tests. With this assumption met, data was evaluated using analysis of variance, ANOVA followed by Dunn's or Bonferroni posthoc test. P-value <0,05 was considered statistically significant. All statistical analyses were performed with Prism 5.0 (GraphPad Software, Inc., La Jolla, CA).

## Supporting Information

Figure S1 **Effect of** ***N. vitripennis*** **venom on cell viability in Raw264.7 cells.** Viability was measured after 6 and 24 hours venom incubation in an MTT assay by adding 3-(4,5-dimethylthiazol-2-yl)-2,5-diphenyltetrazolium bromide (MTT) solution (0,5mg/ml) to the cells. This solution was incubated at 37°C until blue deposits were visible. The formazan crystals were then solubilized in SDS/HCl solution and incubated for 5 hours at 37°C. The absorbance was determined colorimetrically at 595 nm. White bars represent 6 hours venom incubation, black bars represent 24 hours venom incubation. Normality was confirmed by a Shapiro-Wilk test (W = 0.9677). * p-value <0.05, ANOVA and Dunn's test.
(TIF)

Figure S2 **Venom from** ***N. vitripennis*** **inhibits TNF-induced expression of a NF-κB-dependent reporter gene: not normalized luciferase and β–galactosidase values.** L929sA cells stably transfected with a NF-κB-dependent reporter gene were pretreated with indicated concentrations of venom for 15 minutes followed by stimulation for 6 hours with TNF (2000 IU/ml). (A) Luciferase activity and (B) β-galactosidase expression. The data are expressed as the mean ±S.D. of three biological replicates. Normality was confirmed by a Shapiro-Wilk test (W = 0.9337 for A; W = 0.9662 for B). * p<0.05, **** p<0.0001 versus TNF alone, ANOVA with Bonferroni posthoc test.
(TIF)

Figure S3 **Venom does not inhibit PMA- or dB-cAMP-induced expression of neither an AP-1-dependent reporter gene, nor a CRE-dependent reporter gene respectively.** On the left, L929sA cells stably transfected with an AP-1-dependent reporter gene were pretreated with indicated concentrations of venom for 15 minutes followed by stimulation for 6 hours with phorbol 13-myristate 12-acetate (PMA) (10 nM). On the right, L929sA cells stably transfected with a CRE-dependent reporter gene were pretreated with indicated concentrations of venom followed by stimulation for 6 hours with N(6),2′-O-dibutyryladenosine 3′:5′ cyclic monophosphate (dB-cAMP) (100 µM). (A) Luciferase activity, (B) β-galactosidase expression and (C) normalized expression. The data are expressed as the mean ±S.D. of three biological replicates. Normality was confirmed by a Shapiro-Wilk test (W = 0.8744 and W = 0.7985 for A left and right respectively; W = 0.9124 and W = 0.9521 for B left and right respectively; W = 0.9195 and W = 0.8226 for C left and right respectively). ANOVA with Bonferroni posthoc test.
(TIF)

Figure S4 **Histograms of Western blots that show the effect of venom on cytosolic protein activity.** Raw264.7 cells were left untreated or were pretreated with 5 µg/ml venom for 15 minutes and then stimulated with 1 µg/ml LPS for the indicated times. Total cell extracts were assayed by Western blot analysis using antibodies against indicated proteins: (A) P-IKKα/β normalized with the unphosphorylated IKKβ, (B) P-p65 normalized with the unphosphorylated p65. Bands of these proteins were quantified and data are expressed in histograms as the mean ±S.D. of three biological replicates. Normality was confirmed by a Shapiro-Wilk test (W = 0.7242 for A; W = 0.8585 for B). Statistics were performed by ANOVA with Bonferroni posthoc test.
(TIF)

Figure S5 **Histograms of Western blots that show the effect of venom on phosphorylation of MAPKs.** Raw264.7 cells were left untreated or were pretreated with 5 µg/ml venom for 15 minutes and then stimulated with 1 µg/ml LPS for the indicated times. Total cell extracts were assayed by Western blot analysis using antibodies against indicated proteins. (A) P-p38 normalized with p38, (B) P-ERK normalized with ERK, (C) P-JNK normalized with JNK. Bands of these proteins were quantified and data are expressed in histograms as the mean ± S.D. of three biological replicates. Normality was confirmed by a Shapiro-Wilk test (W = 0.7085 for A; W = 0.8703 for B; W = 0.9012 for C). * p<0.01 versus TNF alone, ANOVA with Bonferroni posthoc test.
(TIF)

Figure S6 **Histograms of Western blots that show the effect of venom on IκBα and A20 protein levels.** Raw264.7 cells were left untreated or were pretreated with 5 µg/ml venom for 15 minutes and then stimulated with 1 µg/ml LPS for the indicated times. Total cell extracts were assayed by Western blot analysis using antibodies against indicated proteins. (A) IκBα normalized with Tubulin- α, (B) A20 normalized with Tubulin-α. Bands of these proteins were quantified and data are expressed in histograms as the mean ±S.D. of three biological replicates. Normality was confirmed by a Shapiro-Wilk test (W = 0.9253 for A; W = 0.7062 for B). * p<0.01, ** p<0.001 versus TNF alone, ANOVA with Bonferroni posthoc test.
(TIF)
