# Expression of Concern: How the venom from the ectoparasitoid Wasp *Nasonia vitripennis* exhibits anti-inflammatory properties on mammalian cell lines

**DOI:** 10.1371/journal.pone.0324681

**Published:** 2025-05-20

**Authors:** The PLOS One Editors

After this article [1] was published, concerns were raised regarding results presented in Figs 3–4, and 6.

**Fig 3:**
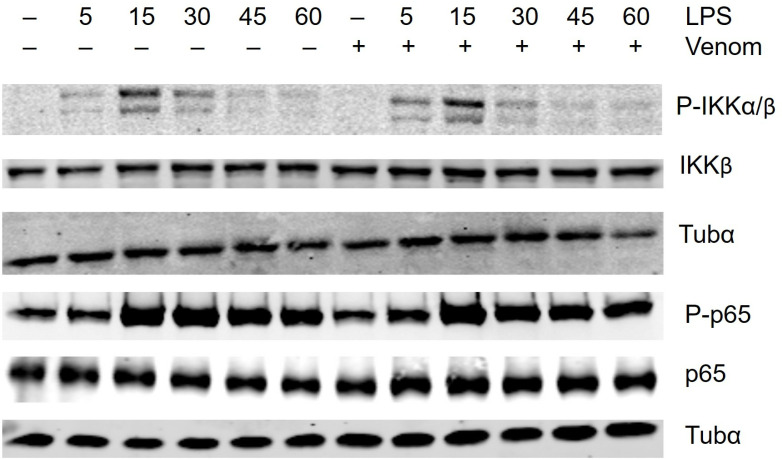
Effect of venom on cytosolic protein activity. Raw264.7 cells were left untreated or were pretreated with 5 µg/ml venom for 15 minutes and then stimulated with 1 µg/ml LPS for the indicated times. Total cell extracts were assayed by Western blot analysis using antibodies against indicated proteins. The unphosphorylated protein and Tubulin-α were used as loading controls. A representative result from three separate experiments is shown.

**Fig 4:**
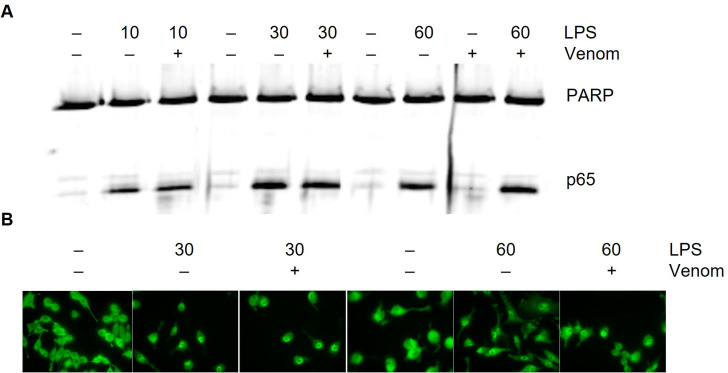
Effect of venom on nuclear translocation of p65. A. Western blot analysis of nuclear extracts from Raw264.7. After pre-incubation with *N. vitripennis* venom (5 µg/ml) for 15 minutes, cells were induced fr the indicated times with LPS (1 µg/ml). Subsequently, nuclear extracts were subjected to Western blot analysis to determine p65 levels. Separation of nuclear and cytoplasmic fractions was verified using PARP as control for the nuclear fractions. B. After 15 minutes pre-incubation with *N. vitripennis* venom, cells were induced for the indicated times with LPS (1 µg/ml). Immunofluorescence staining was performed to visualize the trafficking of the p65 subunit. Representative results from three separate experiments are shown.

Specifically:

- The Fig 3 Tubα panel for P-p65 and p65 appears similar to the Fig 6 Tubα panel for P-p38 and p38.
- In Fig 4A:
  - Lanes 7 and 8 appear similar in the p65 panel.
  - There appear to be vertical discontinuities between lanes in both panels.

The corresponding author stated that the Tubα panel described above in Figs 3 and 6 is the same image, as the Fig 3 P-p65 panel and Fig 6 P-p38 panel were run on the same blot therefore the same loading control was used. They stated that the Tubα panel for P-IKKα/β and IKKβ is incorrect and provided an updated version of Fig 3 with a corrected Tubα blot for P-IKKα/β and IKKβ. They also stated that all panels in Fig 3 were run on different blots.

Additionally, the corresponding author provided an alternative version of Fig 6 (S5 File) showing an additional Tubα panel for ERK originating from the same blot, and updated panels for p38 and for JNK and the associated Tubα panel. The corresponding author stated that the underlying data for the originally published p38 panel, and the Tubα loading control for P-JNK and JNK, are no longer available. They stated that in the updated Fig 6 (S5 File), the Tubα loading controls for P-p38, p38, and JNK were detected on different blots to P-p38, p38 and JNK but on the same blot to P-JNK. Upon editorial review, it was noted that in the updated Fig 6 (S5 File), the Tubα loading control for P-JNK and JNK appear to be detected on a different blot to P-JNK and JNK.

The corresponding author provided the underlying blot data for Figs 3 and 6 (S1 and S3 Files). Upon editorial review of this underlying data, it was noted that for each protein of interest, the phosphorylated and unphosphorylated proteins were detected on separate blots. In light of this observation, the *PLOS One* Editors advise readers to interpret the results and conclusions pertaining to the effects of LPS and *N. vitripennis* venom on the NF-κB and ERK pathway proteins with caution.

The corresponding author provided the underlying data for Fig 4A (S2 File) and stated that the panels were spliced from a western blot that contained additional lanes. The corresponding author indicated that multiple lanes in the published Fig 4A were labelled incorrectly. Upon editorial review of these underlying data, it was noted that lane 7 in the p65 panel was included in error. Fig 4A is updated here to provide the original blot without splicing and with the lanes labelled correctly.

The corresponding author states that the underlying data for all other results in [1] not provided with this notice are available upon request.

The *PLOS One* Editors issue this Expression of Concern to notify readers of the above concerns and to relay the underlying data provided.

## Supporting information

S1 File Original data underlying Fig 3. (ZIP)

S2 File Original data underlying Fig 4A. (ZIP)

S3 File Available original data underlying Fig 6 and replicate data from the time of the original experiments. (ZIP)

S4 File Original data underlying Fig 8. (TIF)

S5 File Alternative version of Fig 6. The updated panels for JNK and the associated Tubα panel, and the p-38 panel are from replicate experiments from the time of the original experiments.
(JPG)

## References

[1] Danneels EL, Gerlo S, Heyninck K, Van Craenenbroeck K, De Bosscher K, Haegeman G, et al. How the venom from the ectoparasitoid Wasp nasonia vitripennis exhibits anti-inflammatory properties on mammalian cell lines. PLoS One. 2014;9(5):e96825. doi: 10.1371/journal.pone.0096825 24821138 PMC4018385

