# Supplementary figures and images for: How the Venom from the Ectoparasitoid Wasp Nasonia vitripennis Exhibits Anti-Inflammatory Properties on Mammalian Cell Lines
Source: PLoS One. 2014 May 12;9(5):e96825. doi: 10.1371/journal.pone.0096825 (PMC4018385; doi:10.1371/journal.pone.0096825)

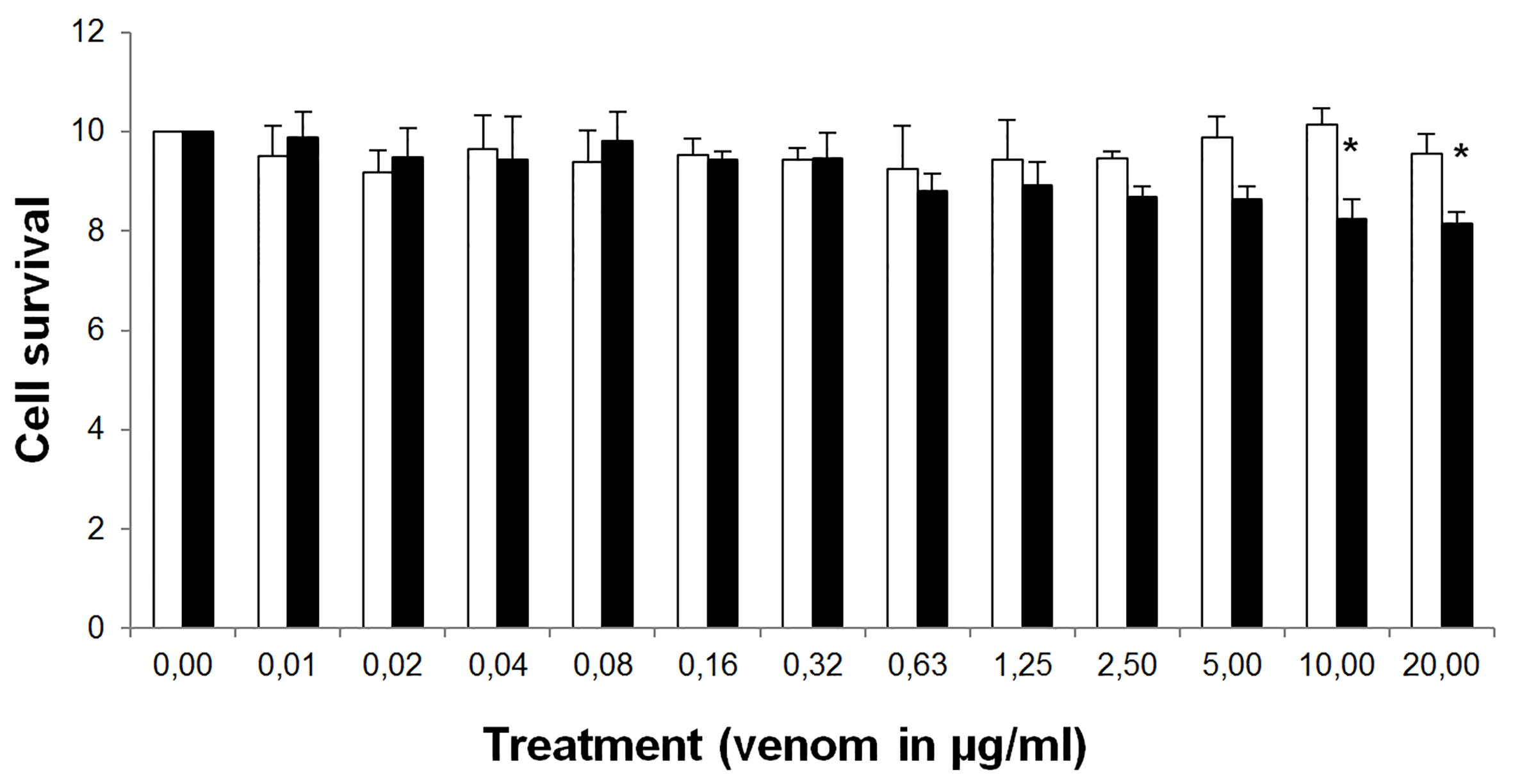

Supplement: Figure S1 — Effect of N. vitripennis venom on cell viability in Raw264.7 cells. Viability was measured after 6 and 24 hours venom incubation in an MTT assay by adding 3-(4,5-dimethylthiazol-2-yl)-2,5-diphenyltetrazolium bromide (MTT) solution (0,5mg/ml) to the cells. This solution was incubated at 37°C until blue deposits were visible. The formazan crystals were then solubilized in SDS/HCl solution and incubated for 5 hours at 37°C. The absorbance was determined colorimetrically at 595 nm. White bars represent 6 hours venom incubation, black bars represent 24 hours venom incubation. Normality was confirmed by a Shapiro-Wilk test (W = 0.9677). * p-value <0.05, ANOVA and Dunn's test. (TIF) [file pone.0096825.s001.tif]

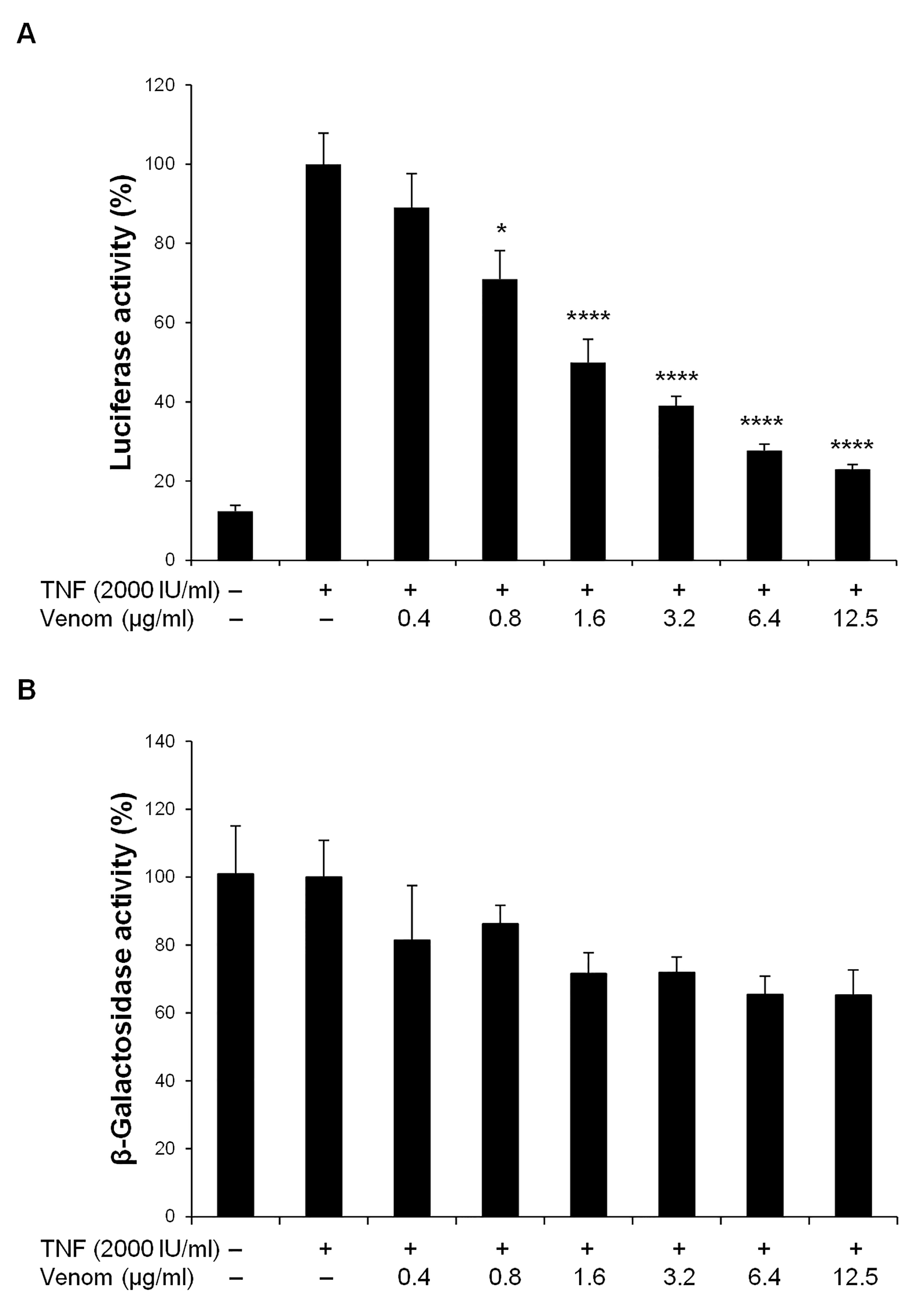

Supplement: Figure S2 — Venom from N. vitripennis inhibits TNF-induced expression of a NF-κB-dependent reporter gene: not normalized luciferase and β–galactosidase values. L929sA cells stably transfected with a NF-κB-dependent reporter gene were pretreated with indicated concentrations of venom for 15 minutes followed by stimulation for 6 hours with TNF (2000 IU/ml). (A) Luciferase activity and (B) β-galactosidase expression. The data are expressed as the mean ±S.D. of three biological replicates. Normality was confirmed by a Shapiro-Wilk test (W = 0.9337 for A; W = 0.9662 for B). * p<0.05, **** p<0.0001 versus TNF alone, ANOVA with Bonferroni posthoc test. (TIF) [file pone.0096825.s002.tif]

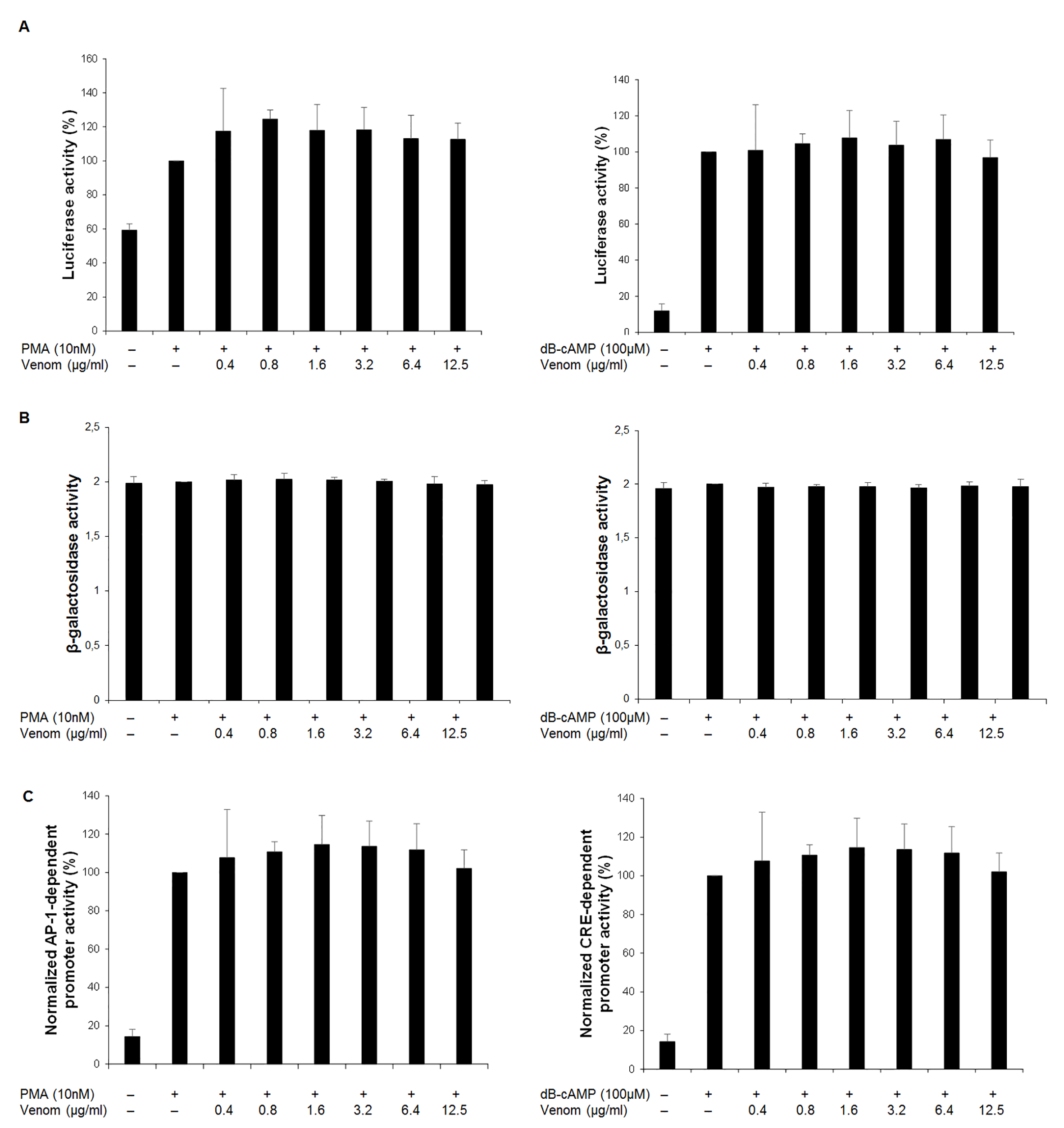

Supplement: Figure S3 — Venom does not inhibit PMA- or dB-cAMP-induced expression of neither an AP-1-dependent reporter gene, nor a CRE-dependent reporter gene respectively. On the left, L929sA cells stably transfected with an AP-1-dependent reporter gene were pretreated with indicated concentrations of venom for 15 minutes followed by stimulation for 6 hours with phorbol 13-myristate 12-acetate (PMA) (10 nM). On the right, L929sA cells stably transfected with a CRE-dependent reporter gene were pretreated with indicated concentrations of venom followed by stimulation for 6 hours with N(6),2′-O-dibutyryladenosine 3′:5′ cyclic monophosphate (dB-cAMP) (100 µM). (A) Luciferase activity, (B) β-galactosidase expression and (C) normalized expression. The data are expressed as the mean ±S.D. of three biological replicates. Normality was confirmed by a Shapiro-Wilk test (W = 0.8744 and W = 0.7985 for A left and right respectively; W = 0.9124 and W = 0.9521 for B left and right respectively; W = 0.9195 and W = 0.8226 for C left and right respectively). ANOVA with Bonferroni posthoc test. (TIF) [file pone.0096825.s003.tif]

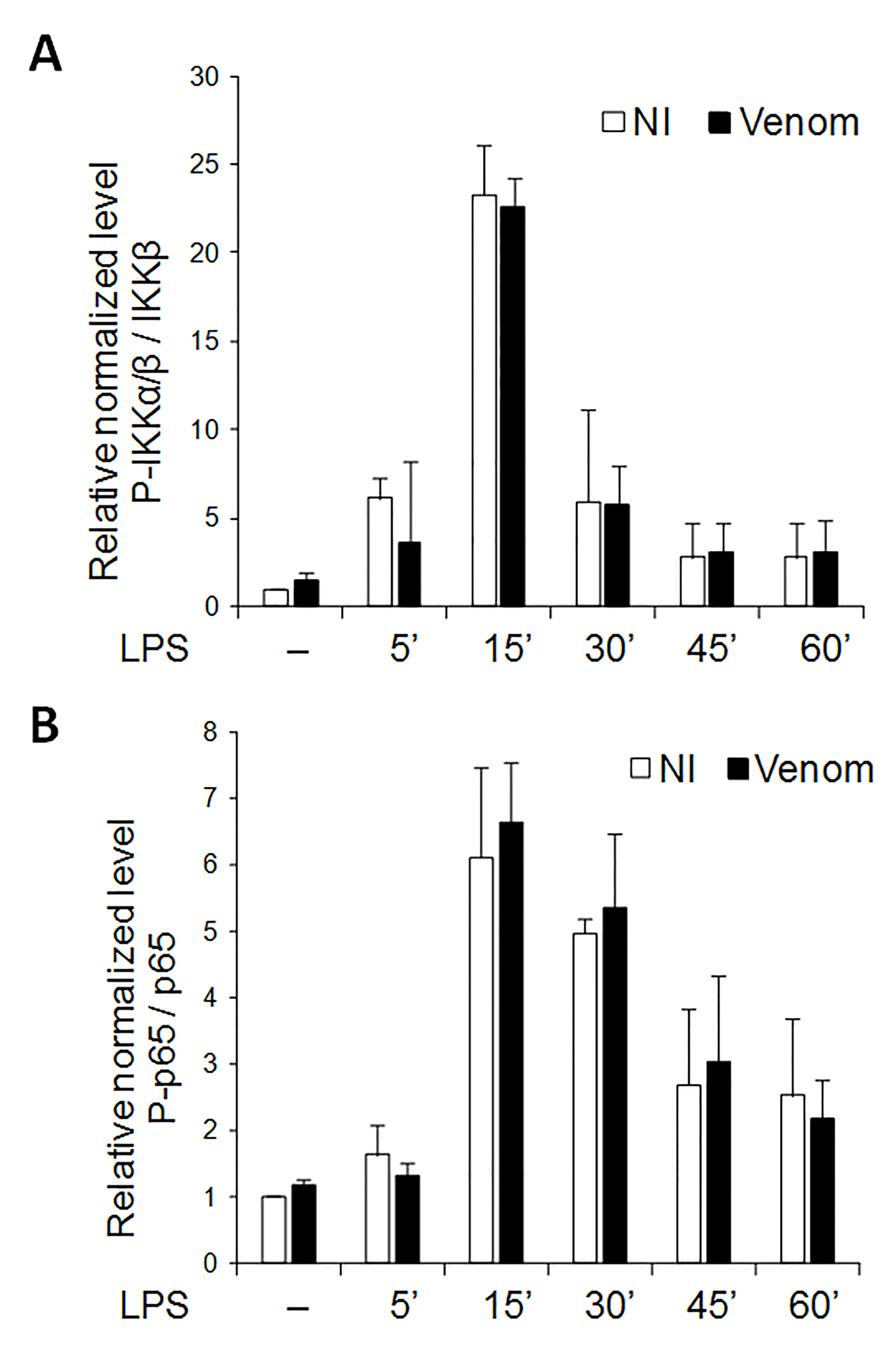

Supplement: Figure S4 — Histograms of Western blots that show the effect of venom on cytosolic protein activity. Raw264.7 cells were left untreated or were pretreated with 5 µg/ml venom for 15 minutes and then stimulated with 1 µg/ml LPS for the indicated times. Total cell extracts were assayed by Western blot analysis using antibodies against indicated proteins: (A) P-IKKα/β normalized with the unphosphorylated IKKβ, (B) P-p65 normalized with the unphosphorylated p65. Bands of these proteins were quantified and data are expressed in histograms as the mean ±S.D. of three biological replicates. Normality was confirmed by a Shapiro-Wilk test (W = 0.7242 for A; W = 0.8585 for B). Statistics were performed by ANOVA with Bonferroni posthoc test. (TIF) [file pone.0096825.s004.tif]

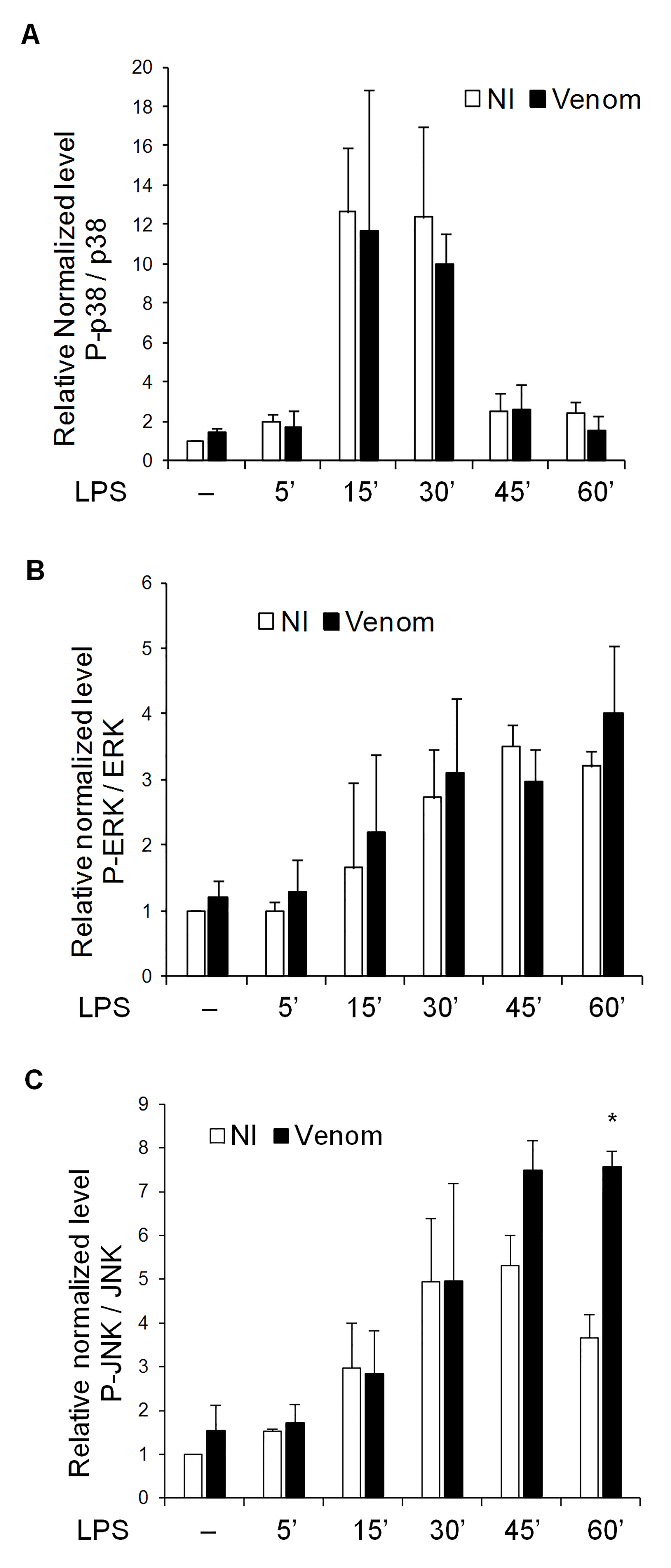

Supplement: Figure S5 — Histograms of Western blots that show the effect of venom on phosphorylation of MAPKs. Raw264.7 cells were left untreated or were pretreated with 5 µg/ml venom for 15 minutes and then stimulated with 1 µg/ml LPS for the indicated times. Total cell extracts were assayed by Western blot analysis using antibodies against indicated proteins. (A) P-p38 normalized with p38, (B) P-ERK normalized with ERK, (C) P-JNK normalized with JNK. Bands of these proteins were quantified and data are expressed in histograms as the mean ± S.D. of three biological replicates. Normality was confirmed by a Shapiro-Wilk test (W = 0.7085 for A; W = 0.8703 for B; W = 0.9012 for C). * p<0.01 versus TNF alone, ANOVA with Bonferroni posthoc test. (TIF) [file pone.0096825.s005.tif]

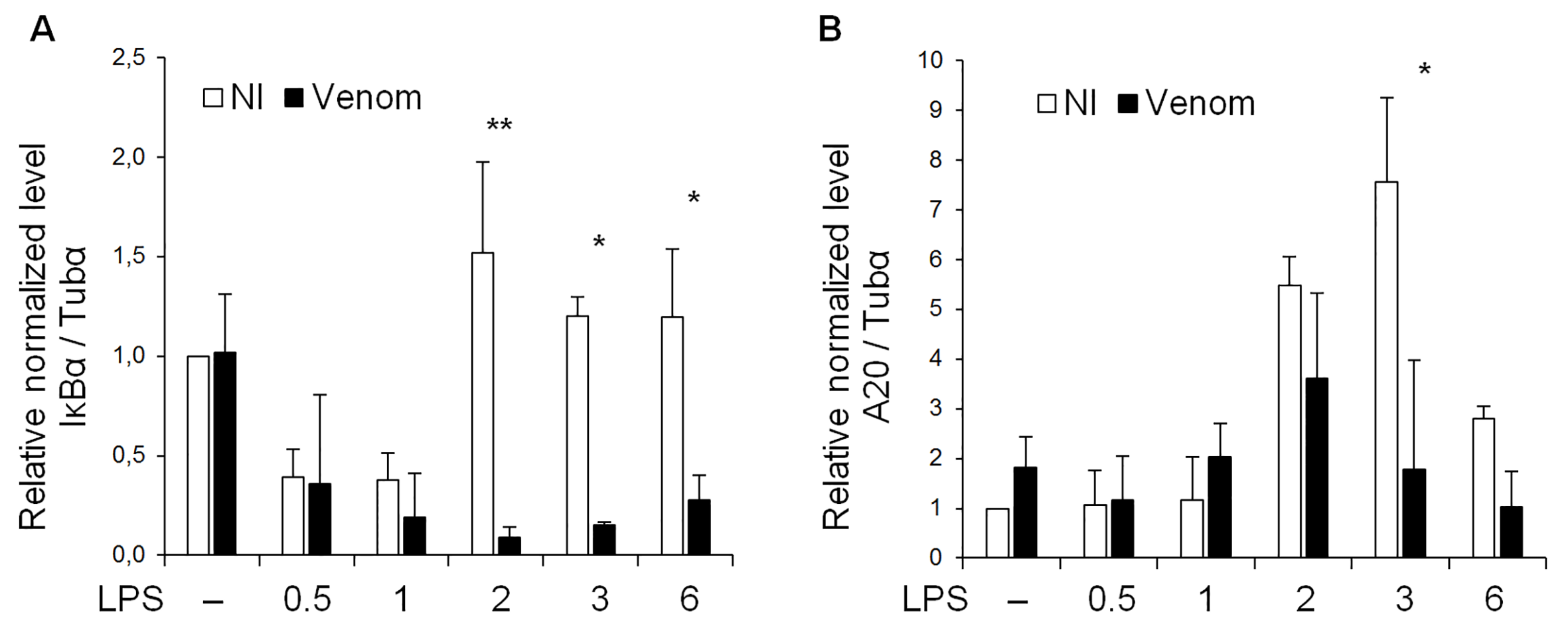

Supplement: Figure S6 — Histograms of Western blots that show the effect of venom on IκBα and A20 protein levels. Raw264.7 cells were left untreated or were pretreated with 5 µg/ml venom for 15 minutes and then stimulated with 1 µg/ml LPS for the indicated times. Total cell extracts were assayed by Western blot analysis using antibodies against indicated proteins. (A) IκBα normalized with Tubulin- α, (B) A20 normalized with Tubulin-α. Bands of these proteins were quantified and data are expressed in histograms as the mean ±S.D. of three biological replicates. Normality was confirmed by a Shapiro-Wilk test (W = 0.9253 for A; W = 0.7062 for B). * p<0.01, ** p<0.001 versus TNF alone, ANOVA with Bonferroni posthoc test. (TIF) [file pone.0096825.s006.tif]
