# Supplementary figures and images for: Expression of Concern: How the venom from the ectoparasitoid Wasp Nasonia vitripennis exhibits anti-inflammatory properties on mammalian cell lines
Source: PLoS One. 2025 May 20;20(5):e0324681. doi: 10.1371/journal.pone.0324681 (PMC12091800; doi:10.1371/journal.pone.0324681)

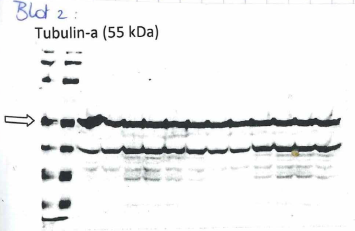

Supplement: S3 File — (ZIP) [file pone.0324681.s003.zip › S3 File/Fig6_2013-07-17-Raw_JNK_Tub.png]

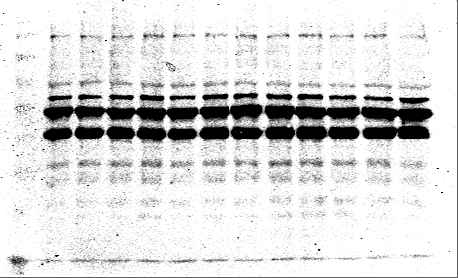

Supplement: S3 File — (ZIP) [file pone.0324681.s003.zip › S3 File/Fig6_2013-07-17_Raw_Tub.tif]

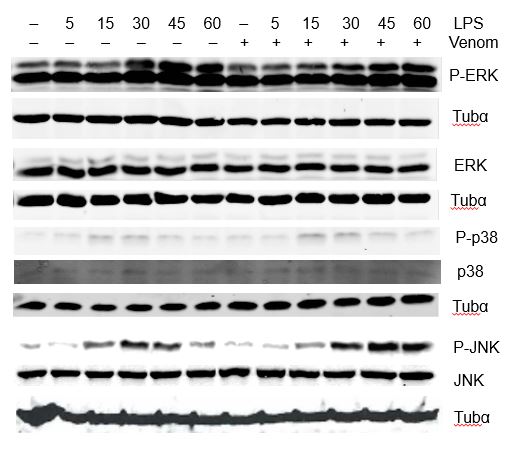

Supplement: S5 File — The updated panels for JNK and the associated Tubα panel, and the p-38 panel are from replicate experiments from the time of the original experiments. (JPG) [file pone.0324681.s005.jpg]
